# Peptide BG From Bitter Gourd (*Momordica Charantia*) Improves Adjuvant-Induced Arthritis by Modulating the Necroptosis/Neutrophil Extracellular Traps/Inflammation Axis and the Gut Microbiota

**DOI:** 10.1155/mi/1995952

**Published:** 2024-12-05

**Authors:** Wenyan Han, Yanan Xu, Suyila Qimuge, Changshan Wang, Xiulan Su

**Affiliations:** ^1^School of Life Science, Inner Mongolia University, Hohhot, Inner Mongolia, China; ^2^Clinical Medical Research Center, Inner Mongolia Bioactive Peptide Engineering Laboratory, The Affiliated Hospital, Inner Mongolia Medical University, Hohhot, Inner Mongolia, China

**Keywords:** gut microbiota, necroptosis, neutrophil extracellular traps, peptide BG, rheumatoid arthritis

## Abstract

**Background:** BG is a novel bioactive peptide derived from bitter gourd (*Momordica charantia*), known for its anti-inflammatory and immunomodulatory properties. In the present study, our objective is to investigate the functional roles and mechanisms of BG in the context of rheumatoid arthritis (RA).

**Methods:** A rat model of adjuvant-induced arthritis (AIA) was established by administering complete Freund's adjuvant (CFA). The viability of BG-mediated AIA was evaluated by assessing changes in rat body weight, joint swelling, ankle joint pathology, inflammation, necroptosis, the formation of neutrophil extracellular traps (NETs), and gut microbiota.

**Results:** The results of the study showed that peptide BG was effective in improving weight loss, joint swelling, serum IgM-rheumatoid factor (IgM-RF) level, and pathological injury of ankle joint in rats with AIA. BG administration resulted in a decrease in erythrocyte sedimentation rate, serum C-reactive protein (CRP), and inflammatory factor (interferon-*γ* (IFN-γ), interleukin-1*β* (IL-1*β*), and tumor necrosis factor-*α* (TNF-*α*)) in AIA rats. Additionally, the administration of CFA resulted in an increase in the protein levels of myeloperoxidase (MPO), neutrophil elastase (NE), citrullinated histone H3 (CitH3), peptidyl arginine deiminase 4 (PAD4), p-mixed lineage kinase domain-like (p-MLKL), and cleaved caspase 8. However, this increase was found to be inhibited by BG treatment. Furthermore, it has been found that peptide BG possesses the capacity to regulate the species composition structure of the intestinal microbiota, thereby, facilitating the reestablishment of microbial diversity and equilibrium.

**Conclusion:** Peptide BG has demonstrated efficacy in ameliorating AIA through its regulation of the necroptosis/NETs/inflammation axis and the gut microbiota. This finding underscores the potential of BG as a promising therapeutic intervention for RA.

**Summary**

• Peptide BG improves adjuvant-induced arthritis (AIA).
• Peptide BG exerts inhibitory effects on the process of necroptosis, thereby, suppressing the formation of neutrophil extracellular traps (NETs) and subsequently mitigating inflammation.
• Peptide BG has the ability to regulate the species composition structure of the intestinal microbiota, thereby, promoting the restoration of microbial diversity and balance.

## 1. Introduction

Rheumatoid arthritis (RA) is a chronic autoimmune disease that predominantly targets the joints, resulting in pain, swelling, and impaired joint function [1]. This condition has a substantial negative impact on the overall well-being and quality of life of affected individuals. While current treatment modalities, including nonsteroidal anti-inflammatory drugs (NSAIDs), disease-modifying antirheumatic drugs, biologics, and immunomodulatory drugs, have demonstrated efficacy in symptom management, they do not provide a definitive cure for the disease and are often accompanied by adverse effects and challenges related to treatment resistance [2]. Therefore, the development of novel and efficacious therapeutic medications assumes paramount significance in the management of RA. The advancement of novel pharmaceuticals holds the promise of enhancing the quality of life for patients, mitigating pain and inflammation, diminishing the likelihood of complications, and facilitating tailored therapeutic approaches [3]. Furthermore, the advancement in pharmaceuticals will propel scientific investigation, yielding novel perspectives on the etiology and mechanisms of RA, and potentially presenting a more sanguine prognosis for individuals afflicted with RA [4]. Consequently, the development of novel and efficacious therapeutic medications holds great importance in enhancing the quality of life for patients suffering from RA, while also contributing to the reduction of healthcare expenses, and the advancement of medical research. This development presents new prospects for progress in the fields of immunology and rheumatology [5].

Peptide BG, which is derived from bitter gourd (*Momordica charantia*), a plant that has long been acknowledged for its medicinal properties in traditional herbal medicine and food, has attracted significant research attention [6]. A study has shown that bitter gourd peptide BG contributes to lowering blood glucose levels, attributed to its enhancement of insulin sensitivity [7]. Additionally, BG-4 can inhibit the inflammatory response induced by lipopolysaccharides [8]. Furthermore, BG-4 has demonstrated its ability to promote apoptosis in human colon cancer cells [9]. Combining these studies, we can speculate that the novel peptide BG might play an anti-inflammatory and cell-regulating role in arthritis, but further experimental validation is still need.

Necroptosis and neutrophil extracellular traps (NETs) are closely associated with inflammation and adjuvant-induced arthritis (AIA), involving the regulation of the immune system and the development of autoimmune diseases. The role of neutrophils in RA is multifaceted, with the formation of NETs being particularly significant in relation to arthritis. Necroptosis is a unique form of cell death associated with various diseases, especially when it leads to an inflammatory response, making its connection with diseases like arthritis even more pronounced [10]. Moreover, not just necroptosis, but other forms of programmed cell death such as apoptosis, autophagy, NETosis, and pyroptosis are also related to the pathogenesis of RA and may become new therapeutic targets in the future [11]. AIA is an experimental model of autoimmune arthritis used to study the mechanisms of autoimmune diseases. In AIA, the immune system erroneously attacks joint tissues, leading to inflammation and joint damage. Research suggests that necroptosis and NETs may play a key role in the development of autoimmune diseases [12]. Molecules released during necroptosis and the presence of NETs may induce inflammatory responses, causing the immune system to mount aggressive reactions against its own tissues [13]. These intertwined processes may be one of the significant factors contributing to the onset of autoimmune diseases such as RA.

The role of the gut microbiota in AIA is an intriguing area of research that underscores the intimate relationship between gut microbes, the immune system, and autoimmune diseases. First, the gut microbiota is crucial in regulating the immune system [14]. Through interactions with the gut immune system, they influence the regulation of immune responses, and gut microbes can modulate the inflammation process [15]. Some microbes may trigger increased inflammation, exacerbating arthritis symptoms. Research suggests that microbes in the gut can affect the severity of arthritis by producing specific metabolites or mediating the activity of immune cells, thereby, influencing the inflammation response [16]. On the other hand, gut microbes can activate pattern recognition receptors in the immune system, such as Toll-like receptors (TLRs). These receptors recognize molecular patterns of microbes, triggering immune responses. Last, the gut microbiota plays a critical role in the integrity of the gut mucosal barrier. Imbalanced microbial composition may lead to a compromised mucosal barrier, allowing inflammatory substances and microbes to enter the bloodstream, initiating a systemic immune response, which may be closely related to the development of arthritis [17]. Although peptide BG has been studied, its role in the field of RA remains insufficiently explored. By revealing the relationships between peptide BG and necroptosis, NETs, inflammation, and the gut microbiota, we can gain a more comprehensive understanding of the potential and scope of this natural bioactive substance. Moreover, this research offers new insights into the treatment of arthritis and has the potential to lay the foundation for personalized treatment.

## 2. Material and Methods

### 2.1. Animals Model and Treatment

All animal procedures in this study were approved by the Ethics Committee of our hospital (YKD2019224) and are in compliance with the Guide for the Care and Use of Laboratory Animals.

The experimental animals (Sprague–Dawley rats, male, aged 8–9 weeks, weighting 180–200 g) were purchased from Vital River Laboratory Animal Technology Co. Ltd. (Beijing, China). They were housed under controlled temperature (25 ± 2°C) and humidity (50%–60%) conditions with a 12-h light/12-h dark cycle and had free access to food and water throughout the experiment. Prior to the experiment, the animals were acclimatized for 5 days. Then, rats were randomly divided into 4 groups (*n* = 8 per group), with the following dosing regimens. The weight of each group of rats on days 1, 4, 10, 17, 24, and 30 were weighed. After treatment, blood, faces, and ankle joints were collected for further measurements.

The timeline of whole animals model and treatment.


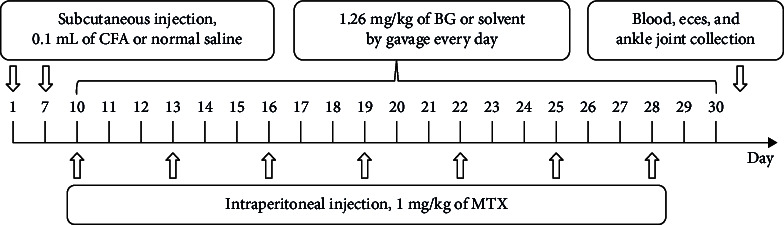


Control group: Rats were only received the vehicle (an equivalent volume of normal saline or corresponding solvent).

AIA group: On the 1st and 7th days, 0.1 mL of complete Freund's adjuvant (CFA, P2036, Beyotime, Shanghai, China) was injected subcutaneously into the footpad of rats.

BG group: From day 10 to day 30, AIA rats were given 1.26 mg/kg of BG (extracted from the entire plant of *M. charantia*; the patent number: ZL201810615496.1) by gavage every day.

Methotrexate (MTX) group: Starting from the 10th day, AIA rats were intraperitoneally injected with 1 mg/kg of MTX (HY-14519, MedChemExpress, Monmouth Junction, NJ, USA) every 3 days [18].

### 2.2. Arthritis Assessment and Paw Swelling Severity

Measurements of ankle joint and paw swelling thickness in rats were taken and recorded. Arthritis score was estimated using a visual score from 0 to 4 (excluding the right rear toe), where score 0 shows no redness or swelling in the paws, score 1 shows erythema and slight swelling in the ankle joint, score 2 shows erythema and slight swelling from ankle to metatarsal or metacarpal joints, score 3 shows erythema and moderate swelling from the ankle joint to the metatarsophalangeal joint or metacarpal joint, and score 4 shows erythema and severe swelling from ankle to toe joint. A score of ≥4 indicates successful modeling [19]. The paw swelling of the experimental rats were estimated at regular time intervals (1, 7, 14, 21, and 30 days) using a vernier caliper via following the previously reported method [20].

### 2.3. Hematoxylin and Eosin (HE) Staining

HE staining was primarily performed using the HE staining reagent kit (G1120, Solarbio, Beijing, China). The ankle joints were preserved in a 10% formalin solution for fixation, and then decalcified using 10% boric acid in a 10% formalin solution and embedded in paraffin. Subsequently, sections were cut at 5 μm thicknesses, deparaffinized, stained with HE, and viewed under a light microscope (Olympus, Japan). The slides were examined for the infiltration of edema, joint cavity space, inflammatory cells, synovial hyperplasia, and cartilage tissue destruction. A histopathologist who was unaware of the experimental conditions classified histopathological changes into normal (score 0), minimal (score 1), mild (score 2), moderate (score 3), and severe (score 4) changes [19].

### 2.4. ELISA

Serum C-reactive protein (CRP; E-EL-R0506c, Elabscience, Wuhan, China), interferon-*γ* (IFN-*γ*; E-EL-R0009c, Elabscience), IgM-rheumatoid factor (IgM-RF; ab178653, Abcam, Cambridge, MA, USA), interleukin-1*β* (IL-1*β*; E-EL-R0012c, Elabscience), tumor necrosis factor-*α* (TNF-*α*; E-EL-R2856c, Elabscience), neutrophil elastase (NE; CSB-E08847r, CUSABIO, Wuhan, China), and myeloperoxidase (MPO; SEKR-0073, Solarbio) levels were measured using corresponding ELISA kits according to the manufacturer's instructions.

### 2.5. Measurement of Erythrocyte Sedimentation Rate (ESR)

The rats were restrained, and the hair near the vein was shaved. The area was disinfected with alcohol, and then the jugular vein was punctured with a sterilized blood collection needle. A 3.8% solution of sodium citrate was used as an anticoagulant, and venous blood was collected into a collection tube at a blood-to-anticoagulant ratio of 4:1. After blood collection, the blood was thoroughly mixed. The collection tube was placed vertically in a sedimentation rack, and after 1 h, the height of the plasma in the upper part of the sedimentation tube was measured in millimeters and recorded.

### 2.6. Western Blot Analysis

Total protein from tissue lysate isolated using RIPA lysis buffer (Beyotime) was loaded on 12% gels (Bio-Rad, Hercules, CA, USA) for SDS–PAGE. After electrophoretic separation, the proteins were transferred onto polyvinylidene difuoride membranes. Subsequently, 5% skimmed milk with TBS (Thermo Fisher Scientific, Waltham, MA, USA) containing 0.1% Tween-20 was used to block the nonspecific protein binding sites. After that, the bands containing target proteins were incubated with primary antibodies overnight at 4°C. The following day, all bands were washed three times and incubated with secondary antibodies for 60 min. Finally, the bands containing target proteins were measured and quantified by the chemiluminescence gel imaging system. Protein expression levels were normalized to the *β*-actin internal control. Primary antibodies included anti-NE (ab314916, 1/1000, Abcam), anticitrullinated histone H3 (anti-CitH3; ab281584, 1/1000, Abcam), antipeptidyl arginine deiminase 4 (anti-PAD4; 17,373-1-AP, 1/1000, Proteintech, Wuhan, China), anti-mixed lineage kinase domain-like (anti-MLKL; ab243142, 1/2000, Abcam), anti-p-MLKL (AF7420, 1/1000, Affinity Biosciences, Jiangsu, China), anti-cleaved caspase 8 (AF5267, 1/1000, Affinity Biosciences), and *β*-actin (66009-1-1 g, 1/10000, Proteintech).

### 2.7. Immunofluorescence Assay

Sections were deparaffinized, and antigen retrieval was performed with a citrate buffer (pH 6.0) in a microwave. Then, sections were permeabilized with 0.2% Triton-X100 and blocked with 3% goat serum. Next, sections were incubated with the first primary antibodies: anti-MPO (22225-1-AP, 1/100, Proteintech), anti-NE (ab314916, 1/50, Abcam),and anti-CitH3 (ab281584, 1/2000, Abcam) in a humidified chamber overnight at 4°C. After washing, sections are stained with DAPI for 10 min in the dark. Finally, sections were mounted using an antifluorescence quenching agent. Sections are examined and imaged with an Olympus inverted fluorescence microscope.

### 2.8. 16S Sequencing and Analysis

The group was divided into four groups: control, CFA, BG.pre (before BG treatment), and BG.post (after BG treatment). Fecal samples were collected from the intestinal contents of rats (five samples per group) in an ultraclean workbench and placed into sterile tubes. Bacterial DNA was extracted using a DNA extraction kit (Fecal DNA Extraction Kit, Qiagen, Hilden, Germany) and subsequently subjected to 16S sequencing [21]. QIIME2 and R package version 3.2.0 were used for the analysis of 16S sequencing data. *α*-diversity indices (Chao1, Shannon, and Simpson) were calculated using QIIME2 to assess species richness and evenness. Rarefaction curves were generated to evaluate the sequencing depth adequacy. Anosim analysis was employed to assess whether intergroup differences were statistically greater than intragroup differences, thereby, determining the significance of groupings. Principal component analysis (PCoA) based on unweighted Unifrac distance was conducted to evaluate differences in species composition between groups. Wilcoxon rank-sum tests were used to identify differentially abundant species at various taxonomic levels between two sample groups. Linear discriminant analysis effect size (LEfSe) was employed to identify intergroup biomarkers (i.e., significantly different species).

### 2.9. Statistical Analysis

Data were expressed as means ± standard deviation (SD). Statistical significance was estimated by one-way analysis of variance (ANOVA) followed by Tukey's post hoc test using GraphPad Prism software (version 8.0). A *p* value <0.05 was considered statistically significant.

## 3. Results

### 3.1. Improvement of CFA-Induced Rat Arthritis by BG

**Figure 1:**
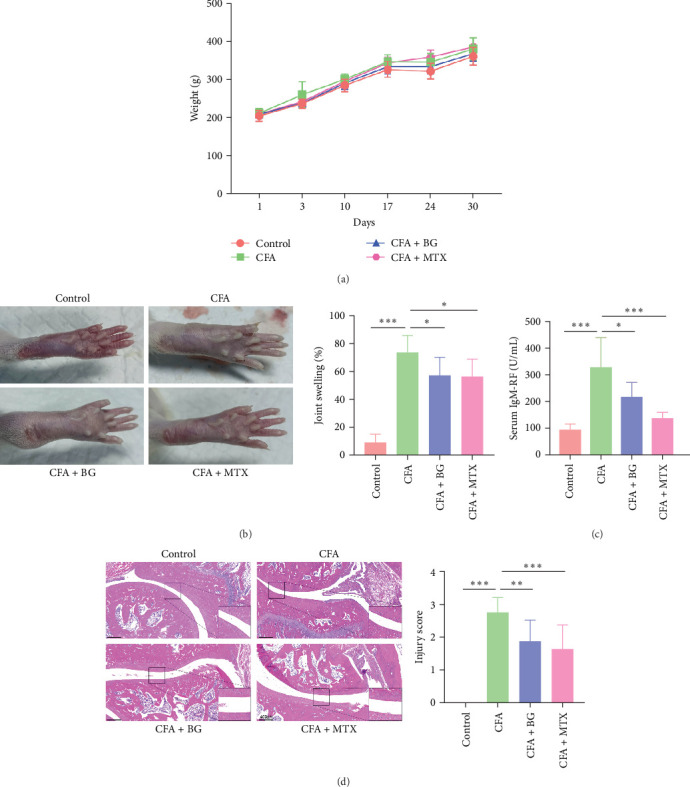
In vivo improvement of adjuvant-induced rat arthritis by BG. (A) Measurement of rat body weight. (B) Evaluation of toe swelling in rats to assess arthritis. (C) ELISA was used to detect serum IgM-RF levels. (D) HE staining was performed to examine pathological changes in ankle joints. All the data are presented as mean ± SD. Statistical differences were analyzed by one-way ANOVA with Tukey's multiple comparisons test. *⁣*^*∗*^*p* < 0.05; *⁣*^*∗∗*^*p* < 0.01; *⁣*^*∗∗∗*^*p* < 0.001. ANOVA, analysis of variance; BG, bitter gourd; CFA, complete Freund's adjuvant; HE, hematoxylin and eosin; IgM-RF, IgM-rheumatoid factor MTX, methotrexate; SD, standard deviation.

The results showed that there was no significant difference in body weight changes among the groups of rats (Figure 1A). CFA–induced modeling resulted in a significant increase in ankle joint swelling (73.88% ± 11.80% vs. control; *p* < 0.001), whereas treatment with BG (57.22 ± 12.97 vs. CFA; *p* < 0.05) or MTX (56.40 ± 12.40 vs. CFA; *p* < 0.05) led to a significant reduction in swelling (Figure 1B). Additionally, CFA–induced modeling increased serum IgM-RF level (328.10 ± 111.76 vs. control; *p* < 0.001), while treatment with BG (217.32 ± 54.19 vs. CFA; *p* < 0.05) or MTX (136.70 ± 22.09 vs. CFA; *p* < 0.001) significantly reduced the IgM-RF level (Figure 1C). In the control group, the joint tissues were intact, and the joint cavity space was normal. In the CFA–induced modeling group, swelling, decreased joint cavity space, inflammatory cell infiltration, synovial hyperplasia, and cartilage tissue damage were observed. Treatment with BG or MTX partially ameliorated the pathological damage (Figure 1D). These results indicated that BG has a significant therapeutic effect on CFA–induced rat arthritis and may serve as a potential treatment for RA.

### 3.2. Anti-Inflammatory Effects of BG on CFA–Induced Arthritic Rats

**Figure 2:**
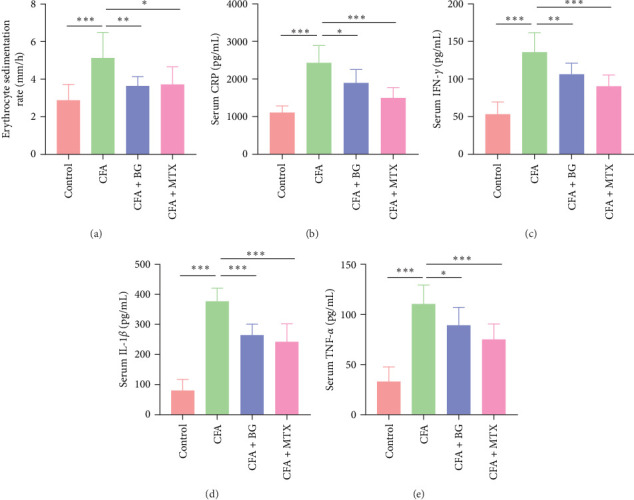
Anti-inflammatory effect of BG on CFA–induced arthritic rats. (A) ESR. (B) ELISA was allowed to detect serum CRP levels. (C–E) The serum levels of IFN-*γ*, IL-1*β*, and TNF-*α* were measured via ELISA. All the data are presented as mean ± SD. Statistical differences were analyzed by one-way ANOVA with Tukey's multiple comparisons test. *⁣*^*∗*^*p* < 0.05; *⁣*^*∗∗*^*p* < 0.01; *⁣*^*∗∗∗*^*p* < 0.001. ANOVA, analysis of variance; BG, bitter gourd; CFA, complete Freund's adjuvant; ESR, measurement of erythrocyte sedimentation rate; IFN-*γ*, interferon-*γ*; IL-1*β*, interleukin-1*β*; MTX, methotrexate; SD, standard deviation; TNF-*α*, tumor necrosis factor-*α*.

Subsequently, we conducted further experiments by measuring various inflammatory factors in the serum and determining the ESR. It was observed that the CFA group of rats exhibited elevated ESR (5.13 ± 1.36 vs. control; *p* < 0.001) and serum CRP (2434.51 ± 460.70 vs. control; *p* < 0.001) levels, indicating the presence of inflammation. However, both BG and MTX significantly reduced the ESR (BG: 3.64 ± 0.50 vs. CFA; *p* < 0.01 and MTX: 3.70 ± 0.95 vs. CFA; *p* < 0.05) and serum CRP (BG: 1903.15 ± 347.31 vs. CFA; *p* < 0.05 and MTX: 1493.04 ± 278.91 vs. CFA; *p* < 0.001) levels in arthritic rats, suggesting the suppression of inflammation (Figure 2A, B). Moreover, CFA–induced modeling led to a significant increase in serum levels of the inflammatory cytokines, including IFN-*γ* (135.38 ± 26.45 vs. control; *p* < 0.001), IL-1*β* (377.46 ± 43.99 vs. control; *p* < 0.001), and TNF-*α* (110.53 ± 18.92 vs. control; *p* < 0.001). BG and MTX effectively inhibited the expression levels of IFN-*γ* (BG: 105.89 ± 15.04 vs. CFA; *p* < 0.01 and MTX: 89.82 ± 15.68 vs. CFA; *p* < 0.001), IL-1*β* (BG: 264.69 ± 36.94 vs. CFA; *p* < 0.001 and MTX: 243.27 ± 59.27 vs. CFA; *p* < 0.001), and TNF-*α* (BG: 89.09 ± 18.10 vs. CFA; *p* < 0.05 and MTX: 75.22 ± 15.17 vs. CFA; *p* < 0.001) in arthritic rats (Figure 2C–E). Taken together, BG has an anti-inflammatory effect in CFA–induced arthritic rats and can exert its therapeutic effects by inhibiting the production of inflammatory factors and reducing tissue inflammation responses.

### 3.3. Regulation of Necroptosis and the Formation of NETs by BG in CFA–Induced Arthritic Rats

**Figure 3:**
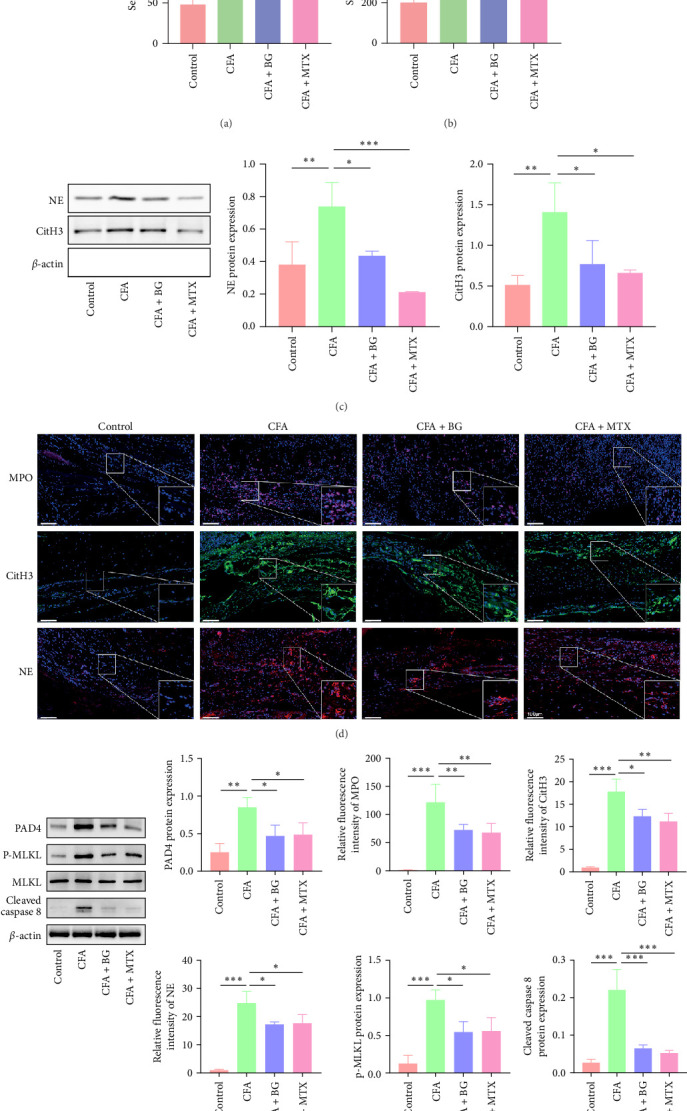
BG regulates necroptosis and NETs formation in CFA-induced arthritic rats. (A, B) The serum levels of MPO and NE were detected by ELISA. (C) Western blot analysis was subjected to measure NE and CitH3 protein levels. (D) Immunofluorescence detection of MPO, NE, and CitH3 levels. (E) The protein levels of PAD4, p-MLKL, MLKL, and cleaved caspase 8 were determined by western blot assay. All the data are presented as mean ± SD. Statistical differences were analyzed by one-way ANOVA with Tukey's multiple comparisons test. *⁣*^*∗*^, *p* < 0.05; *⁣*^*∗∗*^, *p* < 0.01; *⁣*^*∗∗∗*^, *p* < 0.001. ANOVA, analysis of variance; BG, bitter gourd; CFA, complete Freund's adjuvant; CitH3, citrullinated histone H3; MLKL, mixed lineage kinase domain-like; MPO, myeloperoxidase; MTX, methotrexate; NE, neutrophil elastase; Nets, neutrophil extracellular traps; PAD4, peptidyl arginine deiminase 4; SD, standard deviation.

Next, we found that modeling elevated serum MPO (116.40 ± 28.63 vs. control; *p* < 0.001) and NE (523.49 ± 159.27 vs. control; *p* < 0.001) levels. However, BG and MTX administration reduced the levels of MPO (BG: 87.32 ± 15.02 vs. CFA; *p* < 0.05 and MTX: 69.12 ± 15.02 vs. CFA; *p* < 0.001) and NE (BG: 380.87 ± 88.64 vs. CFA; *p* < 0.05 and MTX: 281.32 ± 54.79 vs. CFA; *p* < 0.001; Figure 3A,B). Moreover, CFA upregulated the expression of NE (0.74 ± 0.15 vs. control; *p* < 0.01) and CitH3 (1.41 ± 0.36 vs. control; *p* < 0.01) in the ankle joints of rats, while BG and MTX administration significantly reduced the expression of NE (BG: 0.44 ± 0.03 vs. CFA; *p* < 0.05 and MTX: 0.21 ± 0.00 vs. CFA; *p* < 0.001) and CitH3 (BG: 0.77 ± 0.29 vs. CFA; *p* < 0.05 and MTX: 0.66 ± 0.04 vs. CFA; *p* < 0.05; Figure 3C). The results of immunofluorescence for MPO (CFA: 121.57 ± 31.92 vs. control; *p* < 0.001, BG: 72.60 ± 9.69 vs. CFA; *p* < 0.01, and MTX: 67.79 ± 16.60 vs. CFA; *p* < 0.01), NE (CFA: 24.75 ± 4.31 vs. control; *p* < 0.001, BG: 17.21 ± 0.94 vs. CFA; *p* < 0.05, and MTX: 17.68 ± 3.01 vs. CFA; *p* < 0.01), and CitH3 (CFA: 17.78 ± 2.80 vs. control; *p* < 0.001, BG: 12.35 ± 1.51 vs. CFA; *p* < 0.05, and MTX: 11.17 ± 1.80 vs. CFA; *p* < 0.05) indicated that BG can exert its therapeutic effects by inhibiting the formation of NETs (Figure 3D). Necroptosis is a specific form of cell death closely associated with inflammatory and autoimmune reactions. CFA led to an upregulation of PAD4 (0.85 ± 0.13 vs. control; *p* < 0.01), p-MLKL (0.97 ± 0.14 vs. control; *p* < 0.001), and cleaved caspase 8 (0.22 ± 0.06 vs. control; *p* < 0.001) protein levels, indicating the activation of necroptosis programs. However, BG and MTX administration partially reversed the effects of CFA on PAD4 (BG: 0.47 ± 0.14 vs. CFA; *p* < 0.05 and MTX: 0.48 ± 0.16 vs. CFA; *p* < 0.05), p-MLKL (BG: 0.54 ± 0.14 vs. CFA; *p* < 0.05 and MTX: 0.56 ± 0.18 vs. CFA; *p* < 0.05), and cleaved caspase 8 (BG: 0.06 ± 0.01 vs. CFA; *p* < 0.001 and MTX: 0.05 ± 0.01 vs. CFA; *p* < 0.001) levels (Figure 3E). Taken together, BG improves CAF–induced necroptosis and the formation of NETs in arthritic rats.

### 3.4. Effects of BG on the Gut Microbiota Species Composition in Arthritic Rats

**Figure 4:**
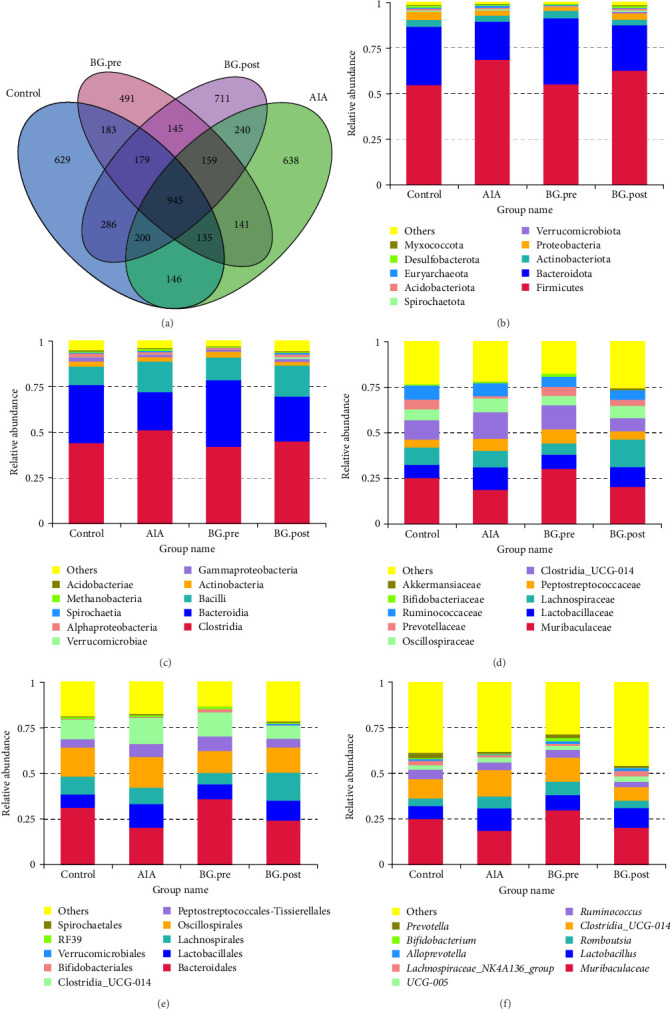
The effect of BG on the species composition structure of gut microbiota in arthritic rats. (A) Statistical analysis of ASVs in each group. (B–F) The relative abundance of species at various taxonomic levels ((B) phylum level, (C) class level, (D) order level, (E) family level, and (F) genus level). Different colors represent different bacterial communities. BG, bitter gourd.

To further investigate the effects of BG on the gut microbiota species composition in arthritic rats, 16S rRNA gene sequencing analysis on fecal samples from the rats was conducted. Statistically analyzing the common and unique ASVs of the four groups, it was found that there were significant changes in the gut microbiota species composition among the groups (Figure 4A). Furthermore, the differences in species abundance of gut microbiota were significantly higher in the BG group than in the CFA group, suggesting that BG can influence the species abundance distribution of gut microbiota in arthritic rats. The species composition of gut microbiota in the BG group of rats differed from that in the CFA group and control group, indicating that BG can influence the species composition of gut microbiota in arthritic rats. Specifically, at the phylum level, *Firmicutes* and *Bacteroidota* were the top two species in relative abundance. The ratio of *Firmicutes* to *Bacteroidota* increased with CAF treatment (ratio: 3.31) compared to the control group (ratio: 1.73), while BG treatment (ratio: 2.54) resulted in a decreased ratio compared to the AIA group (Figure 4B). At the class level, the top three in relative abundance were Clostridia, Bacteroidia, and Bacilli. At the family level, Muribaculaceae was the most abundant species in all groups. At the order level, Bacteroidales was the most abundant species in all groups. At the genus level, *Muribaculaceae* and *Lactobacillus* were the top two species in relative abundance in all groups (Figure 4C–F). Moreover, species with high relative abundance in the AIA group compared to the control group, BG.post group compared to the AIA group, and BG.pre compared to BG. Post group underwent changes. These results suggested that BG can exert its therapeutic effects by modulating the species composition of gut microbiota in arthritic rats.

### 3.5. Effects of BG on the Gut Microbiota Diversity in Arthritic Rats

**Figure 5:**
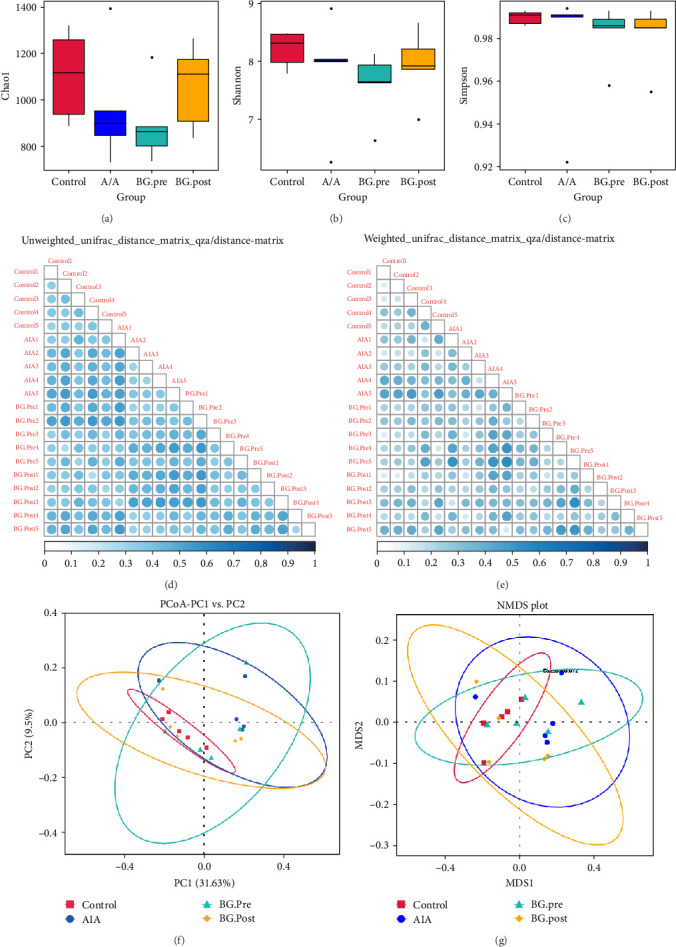
The impact of BG on the gut microbiota diversity in arthritic rats. (A–C) *α*-diversity calculated from the 16S rRNA gene sequencing data using Chao1 (A), Shannon (B), and Simpson (C) indices, respectively. (D–E) Species diversity analysis. The size and color of the dots represent the difference coefficient between the two samples. The larger the dot, the darker the corresponding color, indicating a greater difference between the two samples. On the contrary, the smaller the dot, the lighter the corresponding color, indicating a smaller difference between the two samples. (F–G) *β*-Diversity analysis, PCoA of the three groups based on unweighted unifrac and weighted unifrac. BG, bitter gourd; PCoA, principal component analysis.

Then, we investigated the effects of BG on the gut microbiota diversity in arthritic rats. The experimental results showed that compared to the control group, the AIA group had a decrease in total species count and diversity, with poorer species evenness. Compared to the AIA group, the BG.post group showed a partial recovery in total species count, with no significant changes in diversity and evenness. In comparison to the BG.post group, the BG.pre group exhibited a decrease in total species count and diversity, with a slight decrease in evenness (Figure 5A–C). There were significant differences in species composition among the groups (Figure 5D, E). Additionally, the gut microbiota *β*-diversity index in the BG group of rats was significantly higher than that in the CFA group and control group, indicating that BG can increase the *β*-diversity of gut microbiota in arthritic rats (Figure 5F, G). These results suggest that BG can influence the species composition of gut microbiota by modulating the contribution of species differences in arthritic rats.

### 3.6. Effects of BG on the Contribution of Gut Microbiota Species Differences in Arthritic Rats

**Figure 6:**
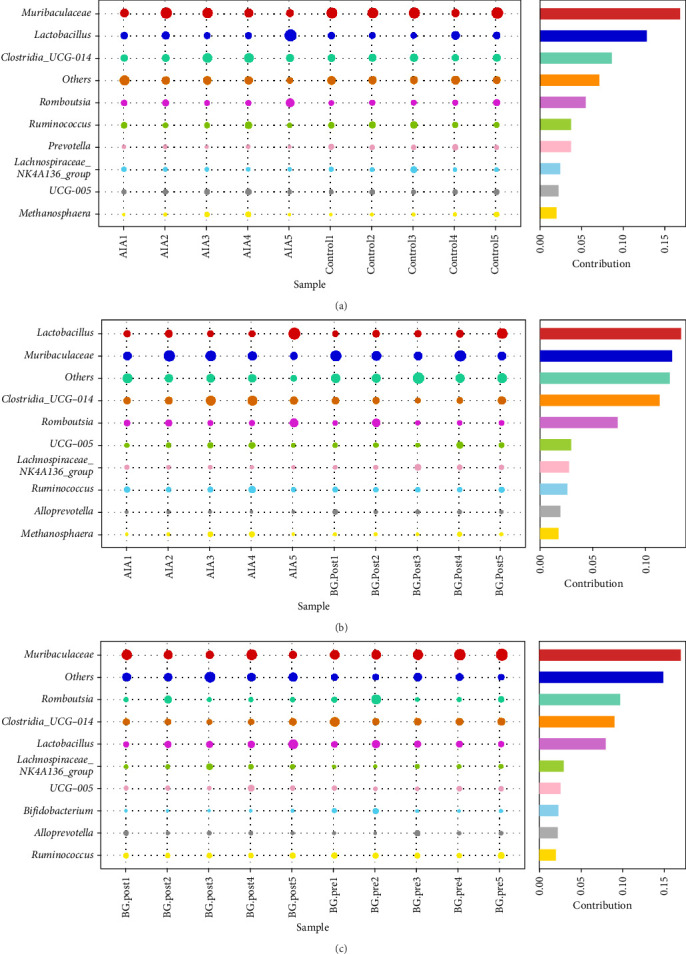
Effects of BG on the contribution of gut microbiota species differences in arthritic rats. (A–C) Simper method quantifying the differential contribution of gut microbiota species between the two groups. The vertical axis represents species at the genus level, the horizontal axis represents samples, the size of the dots represents the relative abundance of the species, and the contribution represents the contribution of the species to the differences between the two groups. BG, bitter gourd.

Further analysis of the contribution of gut microbiota differences revealed that among the control group and the AIA group, the top-ranking species at the genus level were as follows: *Muribaculaceae*, *Lactobacillus*, *Clostridia_UCG_014*, *Romboutsia*, *Ruminococcus*, *Prevotella*, *Lachnospiraceae_NK4A136_group*, *UCG_005*, and *Methanosphaera* (Figure 6A). Furthermore, between the AIA group and BG. Post group, the top-ranking species at the genus level were as follows: *Lactobacillus*, *Muribaculaceae*, *Clostridia_UCG_014*, *Romboutsia*, *UCG-005*, *Lachnospiraceae_NK4A136_group*, *Ruminococcus*, *Alloprevotella*, and *Methanosphaera* (Figure 6B). Between BG.pre and BG.post, the top-ranking microorganisms at the genus level were as follows: *Muribaculaceae*, *Romboutsia*, *Clostridia_UCG_014*, *Lactobacillus*, *Lachnospiraceae_NK4A136_group*, *UCG_015*, *Bifidobacterium*, *Alloprevotella*, and *Ruminococcus* (Figure 6C). In conclusion, the experimental results indicate that BG can influence the species composition of gut microbiota by modulating the contribution of species differences in arthritic rats.

### 3.7. the Impact of BG on Gut Microbiota Species Abundance Differences in Arthritic Rats

**Figure 7:**
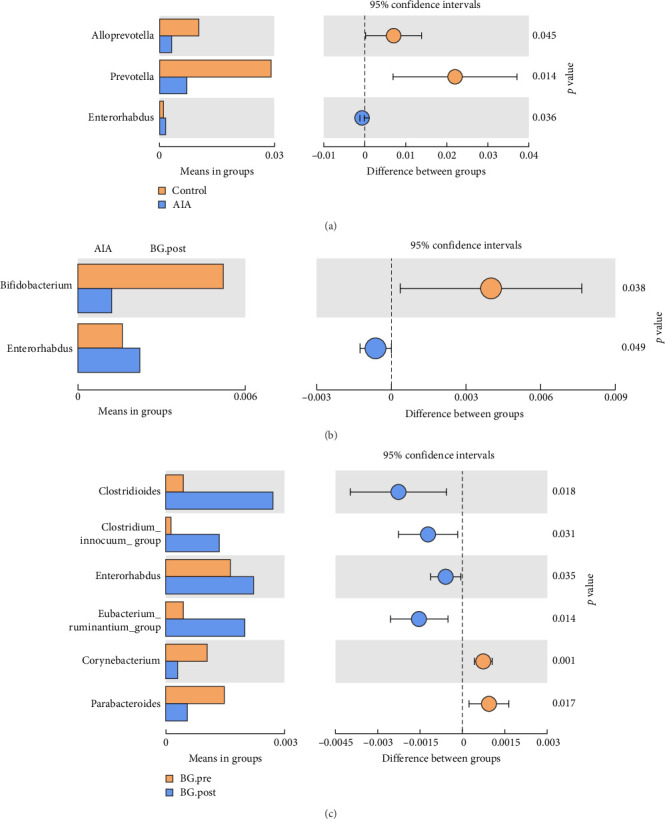
The impact of BG on gut microbiota species abundance differences in arthritic rats. (A–C) The results of Welch's *T*-test, which was used to identify significantly different species between control group and AIA group, AIA group, and BG.post group, as well as BG.pre group and BG.post group. AIA, adjuvant-induced arthritis; BG, bitter gourd.

The experimental results indicate that BG can influence the species abundance differences of gut microbiota in arthritic rats, thereby, affecting the species composition of the gut microbiota. These results suggest that, compared to the control group, the relative abundance of *Alloprevotella* and *Prevotella* in the AIA group significantly decreases, while the abundance of *Enterorhabdus* increases (Figure 7A). Further analysis indicates that BG can affect the species composition of gut microbiota in arthritic rats by modulating differences in species abundance. In comparison to the AIA group, the BG.post group shows a decrease in the abundance of *Bifidobacterium* and an increase in Enterorhabdus (Figure 7B). More specifically, relative to the BG.pre group, the BG.post group exhibits an increase in the abundance of genera such as *Clostridiodes*, *Clostridium*_*innocuum_group*, *Enterorhabdus*, and *Eubacterium_ruminantium_group*, while *Corynebacterium* and *Parabacteroides* exhibit decreased abundance (Figure 7C). These findings indicate that BG can influence the species abundance differences of gut microbiota in arthritic rats, thereby, affecting species richness.

### 3.8. the Impact of BG on Signature Microorganisms of Gut Microbiota in Arthritic Rats

**Figure 8:**
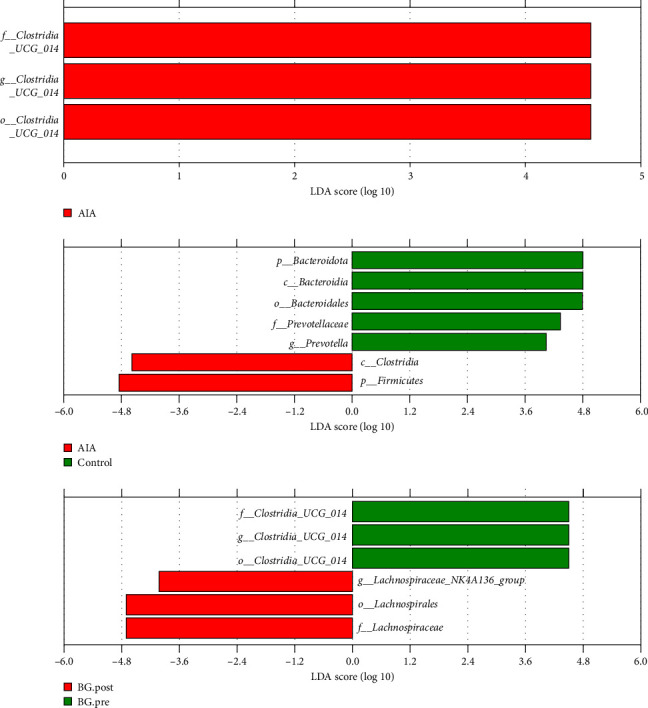
The impact of BG on signature microorganisms of gut microbiota in arthritic rats. Identification of the signature microbiota at the genus level using LEfSe in each group. The length of the bars represents the magnitude of the effect of significantly different species (LDA score), where an absolute value greater than the preset threshold (default: 2) indicates a statistically significant differential marker species. LDA score chart displays the significant differences among the bacterial genera. Columns of different colors represent different groups, and their lengths indicate the LDA score magnitude, reflecting the importance of differences in bacterial genera. BG, bitter gourd; LEfSe, linear discriminant analysis effect size.

Finally, we explored the functional role of BG and found that it can exert its therapeutic effects by modulating the relative abundance of gut microbiota signature organisms in arthritic rats. Experimental results showed that when comparing the control group to the AIA group, the signature organism in the control group was *Prevotella*. When comparing the AIA group to the BG.post group, the signature organism in the AIA group was *Clostridia_UCG_0014*. When comparing the BG.post group to the BG.pre group, the signature organism in the BG.pre group was *Clostridia_UCG_0014* (Figure 8), all of which are pro-inflammatory bacteria. These results suggest that BG can modulate the relative abundance of gut microbiota signature microorganisms in arthritic rats, thereby influencing the microbial community.

## 4. Discussion

RA is a common autoimmune disease characterized by joint pain, swelling, and stiffness [22]. Currently, the primary treatment for RA involves the use of immunosuppressants and NSAIDs to alleviate symptoms. However, these treatment methods come with certain side effects and do not provide a complete cure for RA [23]. Therefore, researching new treatment approaches holds significant importance for improving the quality of life for RA patients, reducing healthcare costs, and advancing medical research in the fields of immunology and rheumatology. In this context, peptide BG, derived from bitter gourd, has sparked widespread research interest. In the present study, BG was proved promising antiarthritic potential mediated, at least in part through inhibiting necroptosis/NETs/inflammation axis and balancing gut microbiota.

Previous studies have shown that CFA can induce ankle joint edema, inflammatory cell infiltration, synovial hyperplasia, and cartilage tissue destruction in rats [24, 25], and BG effectively improved these CFA–induced arthritis lesions. The rat model of arthritis also exhibited significant inflammatory response, and peptide BG can significantly inhibit serum pro-inflammatory factor levels, including IgM-RF, CRP, IL-1*β*, IFN-*γ*, and TNF-*α* [26]. In innate immune and inflammatory responses, neutrophils form NETs structures by expelling contents such as MPO, NE, and CitH3 [27]. A study suggested that NETs, through the release of DNA and proteins, promote inflammatory and autoimmune responses, thereby, contributing to the onset and progression of RA [28]. Additionally, PAD4, an enzyme that promotes the formation of NETs, play crucial roles in RA [29]. Peptide BG can inhibit the expression of PAD4, thereby, reducing the release of NETs and the occurrence of inflammatory responses.

Necroptosis is a tightly regulated inflammatory necrotic cell death signaling pathway. Studies have shown that necroptosis is involved in autoimmune diseases, such as RA [30]. Necroptosis can control the generation of NETs and mediate complement activation, endothelial damage, and autoimmune vasculitis [12]. MLKL is a key pathway protein in necroptosis, and the RIPK1/RIPK3/MLKL pathway is involved in the formation of PAD4-dependent NETs in neutrophils [31]. The results of this study confirmed that BG administration can inhibit the RIPK1/RIPK3/MLKL pathway and suppress the expression of downstream cleaved caspase 8. These findings demonstrated that peptide BG may inhibit the formation of NETs by suppressing the process of necroptosis, thus reducing inflammatory reactions and joint pain.

Furthermore, there is a close relationship between gut microbiota and the pathogenesis of RA. Studies suggest that gut microbiota dysbiosis can lead to abnormal activation of the immune system and the occurrence of inflammatory responses, thereby, promoting the development of RA [15, 32]. Therefore, regulating the balance of the gut microbiota can become a novel approach to treating RA. Our results demonstrated that peptide BG can regulate the species composition structure of intestinal microbiota and promote the restoration of microbial diversity. Compared to the control group, the abundance of *Alloprevotella* and *Prevotella* in the gut microbiota of the model group rats significantly decreased, while *Enterorhabdus* increased. BG can affect *α*/*β*-diversity and lead to a decrease in *Bifidobacterium* abundance and an increase in *Enterorhabdus* abundance in the gut microbiota of arthritis rats. This study is based on LEfSe to screen out each group of marker microorganisms and determine *Alloprevotella* and *Clostridia_UCG_0014* can serve as a marker microorganism.

*Alloprevotella* is reduced in patients with RA, and its abundance is significantly correlated with the levels of inflammatory factors in the body [33]. *t*-test method is used to compare different species between the control group and the model group. We found that *Alloprevotella* was decreased in the model group, and this result suggested that the decrease in *Alloprevotella* may be closely related to the pathogenesis of arthritis.

*Bifidobacterium* is a gram-positive bacterium that is generally beneficial to the body. The reduction in *Bifidobacterium* abundance caused by BG administration after modeling may be related to an increase in *Enterorhabdus*. This study hypothesizes that there may be a potential interaction between *Bifidobacterium* and *Enterorhabdus*, and the peptide BG could improve arthritis by regulating the ratio of the two bacteria and affecting the content of metabolites such as butyrate.

*Prevotella* has a dual impact on organisms; it facilitates the breakdown of proteins and carbohydrates in the body, but can also promote the development of inflammation. Its involvement in metabolic functions is closely linked to various inflammatory diseases, such as periodontitis, intestinal inflammation, and RA. This study identified a decrease in the abundance of *Prevotella* in rats with CFA–induced arthritis, and its relative abundance can serve as a biomarker for the control group to distinguish CFA-modeled rats. *Prevotella* can synthesize butyrate and propionate from substrates in the body. In arthritis, a reduction in *Prevotella* suggests a decrease in short-chain fatty acids like butyrate, thereby, promoting the disruption of the intestinal barrier. The decrease in *Prevotella* seems to explain the increase in *Enterorhabdus* in rats with arthritis. Studies have confirmed a negative correlation between *Enterorhabdus* and butyrate. Therefore, the reduction in metabolites caused by the decrease in *Prevotella* can promote the increase in *Enterorhabdus* [34, 35].

*Clostridia_UCG_0014* is a pro-inflammatory bacterium, and it is suggested that the broader Clostridia class to which it belongs can release toxins targeting intestinal epithelial cells, leading to the disruption of the intestinal barrier and severe inflammatory responses [36].

The significant enrichment of this bacterium in the model group suggests its close association with CFA–induced arthritis. This study hypothesizes that CFA disrupts the balance of the gut microbiota in model rats, leading to an increase in the abundance of *Clostridia_UCG_0014*, thereby, exacerbating subsequent related inflammation. Considering the result that *Clostridia_UCG_0014* is a marker for the BG.pre group when comparing the BG.post group to the BG.pre group, this study speculates that pretreatment with BG may have limited inhibitory effects on the enrichment of *Clostridia_UCG_0014*.

As a specialized dietary supplement, the recommended daily dosage of BG for humans is 12 mg per 60 kg of body weight. We converted this dosage for rats to facilitate our animal experiments. Therefore, a limitation of this study is that only a single dose of peptide BG was tested, which restricts the ability to assess the full dose–response relationship. Future studies should explore different dosages to determine whether increasing or varying the dose could enhance the therapeutic effects. Additionally, long-term studies are needed to evaluate sustained efficacy and potential side effects of peptide BG treatment.

## 5. Conclusions

Collectively, these findings suggest that peptide BG can effectively improve AIA by regulating the necroptosis/NETs/inflammation axis and the balance of gut microbiota. This study provides valuable insights into the potential benefits of BG in treating RA and highlights the importance of gut microbiota in the pathogenesis of the disease.

## Data Availability

All the data generated or analyzed during this study are included in this published article. The data that support the findings of this study are openly available in “zenodo” at https://sandbox.zenodo.org/uploads/68639.

## Nomenclature

BG: Bitter gourd
RA: Rheumatoid arthritis
AIA: Adjuvant-induced arthritis
CFA: Complete Freund's adjuvant
NETs: Neutrophil extracellular traps
IgM-RF: IgM-rheumatoid factor
CRP: C-reactive protein
NSAIDs: Nonsteroidal anti-inflammatory drugs
TLRs: Toll-like receptors
HE: Hematoxylin and eosin
ESR: Measurement of erythrocyte sedimentation rate
PCoA: Principal component analysis
LEfSe: Linear discriminant analysis effect size
MTX: Methotrexate
MPO: Myeloperoxidase
IFN-*γ:*: Interferon-*γ*
IL-1*β:*: Interleukin-1*β*
TNF-*α:*: Tumor necrosis factor-*α*
NE: Neutrophil elastase
CitH3: Citrullinated histone H3
PAD4: Peptidyl arginine deiminase 4
MLKL: Mixed lineage kinase domain-like
SD: Standard deviation.
